# Sleep and epilepsy: A snapshot of knowledge and future research lines

**DOI:** 10.1111/jsr.13622

**Published:** 2022-04-29

**Authors:** Lino Nobili, Birgit Frauscher, Sofia Eriksson, Steve Alex Gibbs, Peter Halasz, Isabelle Lambert, Raffaele Manni, Laure Peter‐Derex, Paola Proserpio, Federica Provini, Al de Weerd, Liborio Parrino

**Affiliations:** ^1^ Child Neuropsychiatric Unit, Istituto G. Gaslini Genoa Italy; ^2^ Department of Neurosciences, Rehabilitation, Ophthalmology, Genetics, Maternal and Child Health (DiNOGMI) University of Genoa Genoa Italy; ^3^ Analytical Neurophysiology Lab, Montreal Neurological Institute and Hospital McGill University Montreal Quebec Canada; ^4^ Department of Clinical and Experiential Epilepsy, UCL Institute of Neurology University College London London UK; ^5^ Department of Neurosciences, Center for Advanced Research in Sleep Medicine, Sacred Heart Hospital University of Montreal Montreal Quebec Canada; ^6^ Szentagothai János School of Ph.D Studies, Clinical Neurosciences Semmelweis University Budapest Hungary; ^7^ Aix Marseille Univ, Inserm, INS, Institut de Neurosciences des Systèmes Marseille France; ^8^ APHM, Timone Hospital, Clinical Neurophysiology Marseille France; ^9^ Unit of Sleep Medicine and Epilepsy, IRCCS Mondino Foundation Pavia Italy; ^10^ Center for Sleep Medicine and Respiratory Diseases, Lyon University Hospital Lyon 1 University Lyon France; ^11^ Lyon Neuroscience Research Center, CNRS UMR 5292/INSERM U1028 Lyon France; ^12^ Department of Neuroscience, Sleep Medicine Centre ASST Grande Ospedale Metropolitano Niguarda Milan Italy; ^13^ Dipartimento di Scienze Biomediche e Neuromotorie Università di Bologna Bologna Italy; ^14^ IRCCS Istituto delle Scienze Neurologiche di Bologna Bologna Italy; ^15^ Stichting Epilepsie Instellingen Nederland Zwolle Netherlands; ^16^ Department of General and Specialized Medicine, Sleep Disorders Center University Hospital of Parma Parma Italy

**Keywords:** arousal, brain plasticity, circadian rhythm, clock genes, cyclic alternating pattern, disorders of arousal, epileptic spikes, epilepsy surgery, insomnia, memory, sleep apnea, sleep hypermotor epilepsy, sleep oscillations

## Abstract

Sleep and epilepsy have a reciprocal relationship, and have been recognized as bedfellows since antiquity. However, research on this topic has made a big step forward only in recent years. In this narrative review we summarize the most stimulating discoveries and insights reached by the “European school.” In particular, different aspects concerning the sleep–epilepsy interactions are analysed: (a) the effects of sleep on epilepsy; (b) the effects of epilepsy on sleep structure; (c) the relationship between epilepsy, sleep and epileptogenesis; (d) the impact of epileptic activity during sleep on cognition; (e) the relationship between epilepsy and the circadian rhythm; (f) the history and features of sleep hypermotor epilepsy and its differential diagnosis; (g) the relationship between epilepsy and sleep disorders.

## 1 INTRODUCTION

Until the 1980s, polysomnography (PSG) was principally used to clarify diagnostic doubts about epilepsy. By exploiting the potential capacity of sleep to activate subtle or muted paroxysmal abnormalities during wakefulness, and to avoid inducing latent seizures by central nervous system stimulants (e.g. bemegride; Bancaud, Talairach, Waltregny, Bresson, & Morel, 1969; Feuerstein, Kurtz, & Rohmer, 1966), clinical environments dedicated to electroencephalography (EEG) were converted into rudimentary and temporary sleep laboratories. However, because the patient had only to fall asleep and reach the deepest non‐rapid eye movement (NREM) sleep stages, recording was limited to 30–60 min or at most an hour and a half to try to capture also a period of rapid eye movement (REM) stage. Even today, especially in the paediatric field, video‐EEGs are generally scheduled in the morning after sleep deprivation in subjects with suspected epilepsy. In effect, why extend PSG monitoring throughout the night if the diagnostic uncertainties of epilepsy may be resolved after a single sleep cycle? Does it make sense to hire and pay a neurophysiology technician for an entire night? What could the complete sleep histogram ever reveal? A portion of stage N3 could be enough even in the case of NREM parasomnias. The tenacious and visionary curiosity of European sleep medicine pioneers went further, allowing us to investigate the mysterious and fruitful intertwining between sleep and epilepsy. This approach has allowed to shed light on unknown neurophysiological mechanisms, consolidate the role of sleep microstructure, and disclose the impact of ictal and interictal manifestations on daytime vigilance, cognitive functions and autonomic balance. Moreover, important clinical findings on sleep‐related epilepsies (SRE) and co‐morbidities with sleep disorders have been highlighted. In this review, we summarize the most stimulating discoveries and insights reached by the “European school.”

## 2 THE EFFECTS OF SLEEP ON EPILEPSY

Sleep has a significant effect on epilepsy, with NREM sleep facilitating and REM sleep inhibiting epileptic activity (Ng & Pavlova, 2013; Shouse, Farber, & Staba, 2000). Evidence for the effects of sleep on epilepsy is not only present for sleep architecture but also for its microstructure. Analysis of the cyclic alternating pattern (CAP), an EEG marker of unstable sleep, has shown that epileptic activity is not uniformly increased during NREM sleep, but that enhanced epileptic activity is associated with CAP A1 subtypes that consist of recurrent EEG bursts of slow‐wave activation (Parrino, Smerieri, Spaggiari, & Terzano, 2000). Subsequently, the role of slow waves known to orchestrate physiological brain rhythms was investigated in epilepsy (Steriade, 2006). Isolated high‐amplitude slow waves were found to be the main driver of interictal epileptic activity during NREM sleep (Frauscher et al., 2015a), likely mediated by EEG hypersynchronization and by the presence of a bi‐stable state typical of slow oscillations (Steriade, 2006). On the other hand, in some epileptic conditions, the distribution of interictal epileptiform discharges (IEDs) follows the dynamics of spindle frequency activity throughout the night (Ferrillo, Beelke, & Nobili, 2000; Zubler, Rubino, Lo Russo, Schindler, & Nobili, 2017). In contrast, it was shown that REM sleep with (phasic) and without (tonic) rapid eye movements has distinct suppressive impact on interictal epileptic activity, with the most inhibiting effect being present during phasic REM sleep where EEG desynchronization is maximum (Campana et al., 2017; Frauscher, von Ellenrieder, Dubeau, & Gotman, 2016). Apart from these well‐documented relationships, the effect of arousals on sleep and epilepsy remains a crucial issue to be investigated. In particular, it has been shown that epileptic activity (Peter‐Derex et al., 2020; Terzano, Parrino, Anelli, & Halasz, 1989), as well as physiological, paraphysiological and pathological motor events share a common trait of arousal‐activated phenomena (Parrino, Halasz, Tassinari, & Terzano, 2006).

In pre‐surgical epilepsy evaluation as currently performed, analysis of sleep plays at most a minor role. However, in a time of increased efforts undertaken to localize the epileptogenic zone in the interictal EEG, it might be particularly beneficial to take advantage of the distinct properties of sleep. Standard EEG shows that spikes (if present) become more focally restricted during REM sleep and more widespread, revealing additional foci during NREM sleep (Ng & Pavlova, 2013; Sammaritano, Gigli, & Gotman, 1991). Furthermore, a study performing electrical source imaging with high‐density EEG in six patients supported the source localizing value of REM spikes over NREM spikes (Kang et al., 2020). Finally, a recent systematic review concluded that spikes occurring during REM sleep correctly localized the epileptogenic zone in 84% of cases and that REM spikes were never false localizing (McLeod, Ghassemi, & Ng, 2020). On the other hand, capitalizing on supervised machine learning techniques, a recent study found that NREM sleep is best to identify the epileptogenic zone for both single features such as spikes or high‐frequency oscillations, a novel marker of the epileptogenic zone (Frauscher et al., 2017) and the multi‐feature approach (Klimes et al., 2019). One explanation that could reconcile both findings is that REM sleep is particularly useful for features relying on EEG desynchronization effects such as increased localization accuracy of spikes, whereas NREM sleep has particular value for features capitalizing on the impact of synchronization. Utilizing strengths of both states of vigilance might aid to further improve localization accuracy.

In clinical practice it is widely accepted that sleep deprivation can provoke seizures and increases the likelihood of finding specific epileptiform abnormalities in the standard EEG, as cortical excitability increases with time awake (Huber et al., 2013). However, this statement is true only when considering specific epilepsy subtypes. It is known that sleep deprivation results more frequently in seizures in case of generalized epilepsies and in particular in juvenile myoclonic epilepsy (Xu et al., 2018). Further, sleep deprivation protocols aiming to provoke specific epileptiform anomalies in standard EEG were most useful in the context of generalized epilepsies (Renzel, Baumann, & Poryazova, 2016). In contrast, its blind use for all types of epilepsy or focal epilepsies added no further value than a subsequent repeated standard EEG. In a systematic review, Rossi et al. identified only five relevant studies based on focal epilepsy; two of the five studies showed no clear relationship between insufficient sleep and seizure risk (Rossi, Joe, Makhija, & Goldenholz, 2020). The only randomized study performed in the epilepsy monitoring unit in 84 pre‐surgical epilepsy patients found no effect of sleep deprivation on seizure occurrence (Malow, Passaro, Milling, Minecan, & Levy, 2002). Interestingly, recent data obtained in focal drug‐resistant epilepsy suggest that increasing sleep duration by 1.6 hr may lower the risk of seizure occurrence by 27% in the following 48 hr (Dell et al., 2021).

## 3 THE EFFECTS OF EPILEPSY ON SLEEP STRUCTURE

Epilepsy is associated with changes in sleep macro‐ and microstructure (Sudbrack‐Oliveira, Lima Najar, Foldvary‐Schaefer, & da Mota Gomes, 2019). These changes are multifactorial, given that epilepsy is not only seizures but rather a complex, multidimensional condition regarding the underlying pathology, neuropsychiatric and sleep co‐morbidities, and effects of pharmacological and non‐pharmacological treatments (Fisher et al., 2014; Liguori, Toledo, & Kothare, 2021; Romero‐Osorio, Gil‐Tamayo, Nariño, & Rosselli, 2018). However, apart from these manifold factors, evidence suggests that epileptic activity has a direct impact on sleep architecture, sleep continuity and sleep oscillations.

Increased wake after sleep onset is the strongest feature observed in patients with epilepsy (PWE; Crespel, Coubes, & Baldy‐Moulinier, 2000; Dell et al., 2021; Parrino et al., 2012; Peter‐Derex et al., 2020), especially during nights with clinical manifestations. It may result in part from the awakening effect of certain seizures, not only generalized tonic–clonic seizures but also focal, and even paucisymptomatic seizures (Awad & Lüders, 2010; Manni et al., 1997; Yildiz, Tezer, & Saygi, 2012). Seizure‐associated changes in sleep parameters also include a decrease in REM sleep quantity and a delay in the first REM sleep episode (Bazil, Castro, & Walczak, 2000; Dell et al., 2021). Sleep architecture disruption is observed at the microstructural level as well. Generalized interictal discharges are associated with alterations in NREM sleep stability as evident by an increased amount of CAP rate and a longer duration of CAP cycles (Terzano, Parrino, Anelli, Boselli, & Clemens, 1992). A direct arousing effect of ictal and interictal activity has been demonstrated in focal drug‐resistant epilepsy using combined intracranial EEG and PSG recordings, which allow to explore the precise temporal relationship between epileptic discharges and arousals (Malow, Bowes, & Ross, 2000; Peter‐Derex et al., 2020; Terzaghi et al., 2008). Epileptic activity also interferes with sleep oscillations. Epileptiform K‐complexes may be observed in patients with generalized idiopathic or focal epilepsy, being considered as a paroxysmal response to arousing stimuli (Halász, Terzano, & Parrino, 2002; Niedermeyer, 2008). A focal deficit in sleep spindles, whose rate is negatively correlated with the spike index, was reported in childhood epilepsy with centrotemporal spikes (Kramer et al., 2021). Such a decrease in spindle activity is also observed in the region surrounding the epileptic focus in patients with drug‐resistant epilepsy. Epileptic activity may also disrupt the orchestration of sleep oscillations, i.e. through abnormal coupling between hippocampal IED and remote cortical spindles (Gelinas, Khodagholy, Thesen, Devinsky, & Buzsáki, 2016). Changes in REM sleep oscillations have been reported too, with a decrease in density, duration and frequency of sawtooth waves in patients with temporal and extratemporal lobe epilepsy (Vega‐Bermudez, Szczepanski, Malow, & Sato, 2005).

Epilepsy‐related alterations in sleep patterns raise a number of considerations. First, it is worth underlining the bi‐directional interaction between sleep instability and epileptic activity. Regardless of the causal relationship, enhanced sleep instability in PWE may also exert a negative impact on autonomic functions increasing the sympathetic tone during sleep (Tobaldini et al., 2013). Second, disruption of sleep architecture may be particularly pronounced in patients with sleep‐related hypermotor epilepsy (SHE; Loddo et al., 2020; Nobili et al., 2005; Parrino et al., 2012). Third, despite the sleep alterations related to epilepsy, not all patients complain of poor sleep quality. As observed in insomnia, sleep misperception occurs frequently in PWE, although the objective–subjective mismatch remains to be explored (Ng & Bianchi, 2014). Finally, most evidence on the influence of epilepsy on sleep has been gathered from single‐night hospital‐based investigations many of which were performed in the epilepsy monitoring unit and not in a controlled sleep laboratory environment. Longitudinal assessment of sleep in PWE (at diagnosis and during follow‐up, taking into account seizure control, co‐morbidities, anti‐seizure medication, etc.) is recommended to disentangle the role of epilepsy from that of confounding factors, and could benefit from ecological sleep studies using home‐based devices.

## 4 EPILEPSY, SLEEP, BRAIN PLASTICITY AND EPILEPTOGENESIS

Spontaneous cortical oscillations during slow‐wave sleep are associated with neuronal plasticity due to rhythmic spike bursts and spike trains fired by thalamic and neocortical neurons during low‐frequency rhythms characterizing this vigilance state. In vivo experimental data have shown that during slow‐wave sleep oscillations, neuroplasticity changes occur at the level of both thalamic and cortical neurons, which progressively enhance their responsiveness (Steriade & Timofeev, 2003). Experimental and human studies have also shown that the same spontaneous synchronized sleep oscillations may develop into paroxysmal epileptic activities (Steriade, Contreras, & Amzica, 1994). Finally, sleep‐related epileptic transformation of physiological networks may underly plastic changes favouring epileptogenesis (Halász & Szűcs, 2020). Indeed, the normal sleep circuitry and sleep‐specific oscillations (spindles, slow waves), hijacked to generate epileptic activity (Beenhakker & Huguenard, 2009; Steriade et al., 1994), may favour epileptogenesis in the most frequent (developmental) epilepsies (Halász, Bódizs, Ujma, Fabó, & Szűcs, 2019). In particular: (a) absence epilepsy with spike and wave discharges exploits the burst‐firing mode of the corticothalamic system during NREM sleep (Beenhakker & Huguenard, 2009; Gloor, 1978; Steriade, McCormick, & Sejnowski, 1993); (b) in mesio‐temporal epilepsy, hippocampal sharp‐wave‐ripples transform to epileptic spikes joining high‐frequency pathological oscillations (Buzsáki, 2015; Frauscher et al., 2015b); (c) in the frame of perisylvian epileptic network centrotemporal spikes may shift into diffuse discharges as previously observed in patients with electrical status epilepticus in sleep (ESES) and Landau–Kleffner syndrome (LKS; Halász et al., 2019; Halász, Bódizs, et al., 2019; Mirandola et al., 2013; Tassinari et al., 2000).

## 5 IEDs DURING NREM SLEEP: IMPACT ON COGNITION

The NREM sleep seems to play a major role in memory and cognition, regulating synaptic homeostasis (Tononi & Cirelli, 2014) and reshaping hippocampal‐neocortical network necessary for long‐term memory consolidation (Born & Wilhelm, 2012; Buzsáki, 2015; Diekelmann, 2014). The fact that NREM sleep may strongly activate IEDs, including those produced by the mesial temporal regions (Lambert et al., 2018), may have consequences on both synaptic plasticity and hippocampal‐neocortical dialogue.

In childhood epilepsies characterized by a strong activation of IEDs during NREM sleep and cognitive alterations (LKS, continuous spike and waves during slow‐wave sleep), an association between cognitive impairment and an altered overnight decrease of slow waves (a sign of altered slow wave homeostasis) has been reported, suggesting that IEDs may prevent the physiological process of synaptic downscaling. This seems to be supported by the improvement of cognitive functions in these patients after recovery of the homeostatic regulation of slow waves (Bölsterli et al., 2011, 2017).

On the other hand, IEDs occurring during sleep have been suggested to disturb the coupling between hippocampal ripples, thalamic spindles and cortical slow waves, necessary for long‐term memory consolidation (Buzsáki, 1989, 2015). Indeed, recent studies, conducted during the presurgical evaluation of drug‐resistant patients with focal epilepsy, showed a link between NREM sleep‐related hippocampal IEDs and the impairment of long‐term memory consolidation (Lambert et al., 2020, 2021). Hippocampal IED density has been shown to be negatively correlated with hippocampal spindle density (Frauscher et al., 2015b). Knowing the role of spindles on cognitive processes (Schabus et al., 2004), IEDs highly associated with spindle frequency time course in different epileptic syndromes of childhood characterized by cognitive dysfunctions (Baglietto et al., 2001; Gibbs, Nobili, & Halász, 2019) play a negative role on cognition (Kramer et al., 2021). Finally, hippocampal IEDs have also been shown to disturb hippocampal‐frontal networks, inducing spindle‐like activity in the frontal region during NREM sleep, REM sleep and wakefulness (Dahal et al., 2019; Gelinas et al., 2016).

## 6 CIRCADIAN RHYTHM AND EPILEPSY

Circadian rhythms are part of the internal 24‐hr daily cycle of nearly all biological functions. Circadian patterns in seizure occurrence have been recognized for centuries. Advances in diagnostic technology including chronic intracranial EEG recordings have confirmed the clinical observation of different temporal patterns of epileptic activity and seizure occurrence over the 24‐hr period (Baud et al., 2018; Ct, Tk, Ft, & Mj, 2015). The diurnal occurrence of seizures is influenced by several factors, including the type of epilepsy (generalized or focal) and the site of seizure onset (i.e. frontal, temporal, etc.; Khan et al., 2018; Spencer et al., 2016). Generalized seizures have a tendency to occur in the morning following sleep. In focal epilepsies, frontal lobe seizures occur predominantly during sleep, while temporal lobe seizures arise mostly in wakefulness (Hofstra et al., 2011). Of note, these studies do not allow evaluation of whether the observed preferred time of occurrence is modulated by behavioural states (wakefulness versus sleep or drowsiness), environmental conditions or independent effects of the endogenous circadian system. While demonstrating circadian patterns of seizures in humans can be challenging, strong evidence supporting a circadian modulation of seizures is derived from animal models, where rigorous study designs are feasible. In a rat model of limbic epilepsy, the presence of a distinct endogenous circadian distribution of seizures, irrespective of the sleep–wake status, has been shown, and the distribution of seizures relative to time of day resembled the one observed in human mesial temporal lobe epilepsy (Quigg, Straume, Menaker, & Bertram, 1998). Variability in cortical excitability across the circadian cycle and following sleep deprivation has also been shown in analyses of transcranial magnetic stimulation. Cortical excitability increases with time awake and appears to vary according to the epilepsy syndrome (Badawy, Curatolo, Newton, Berkovic, & Macdonell, 2006), but is also modulated by the circadian phase with lower cortical excitability in the evening hours (Ly et al., 2016). Some studies have tried using melatonin to influence circadian rhythm and thereby improve seizure control; however, results have been variable and the role of melatonin in reducing seizures is uncertain (Brigo, Igwe, & Del Felice, 2016). Core circadian genes, *BMAL1* and *CLOCK*, which code for transcription factors, have been shown to influence excitability and seizure threshold (Gerstner et al., 2014; Li et al., 2017). *CLOCK* and *BMAL1* are also involved in the regulation of the mTOR pathways consistent with the notion that mTOR and the circadian system interact to promote epilepsy (Lipton et al., 2015; Zhang et al., 2009). Several regulator proteins bind to a complex GATOR1 to repress the activity of the mTOR‐system. Among them, *DEPDC5*, *NPRL2* and *NPRL3* are interesting, as mutations in these genes are specifically associated with SHE (Ricos et al., 2016; Scheffer et al., 2014). Further studies are needed to clarify the relationship, but this may inspire alternative future treatment options, including gene therapy or optogenetics. Variability in circadian seizure preponderance also opens the possibility for chronotherapy. An obvious treatment strategy in epilepsy to date is to treat at times of greatest occurrence of seizures based on historically highest seizure or epileptogenicity levels in relation to wakefulness, sleep, circadian or non‐circadian rhythms (Ramgopal, Thome‐Souza, & Loddenkemper, 2013). Such personalized antiepileptic drug‐dosing regimens may improve seizure control and reduce side‐effects as well as risks associated with seizures.

## 7 FROM NOCTURNAL FRONTAL LOBE EPILEPSY (NFLE) TO SHE

The first description of SHE dates back to 1981, when Lugaresi and Cirignotta described five patients presenting bizarre motor behaviours or sustained dystonic postures during sleep. They named this condition “hypnogenic paroxysmal dystonia” and later “nocturnal paroxysmal dystonia” (NPD) to emphasize the complex, violent, dystonic and ballistic features of the episodes (Lugaresi & Cirignotta, 1981). In 1990, Tinuper *et al*. confirmed the epileptic nature of NPD documenting clear‐cut epileptic EEG abnormalities in three patients with NPD (Tinuper et al., 1990). The term “NFLE” was coined, defining NFLE as a syndrome characterized by a spectrum of motor manifestations of varying complexity and duration from the shortest episodes (paroxysmal arousals, PA) to the most prolonged events (epileptic nocturnal wanderings, ENW; Montagna, 1992; Plazzi, Tinuper, Montagna, Provini, & Lugaresi, 1995; Provini, Plazzi, & Lugaresi, 2000). A video‐PSG study of 100 consecutive NFLE cases highlighted that seizure frequency was high with frequent clustering, and that PA, NPD and ENW could occur in the same patient representing the continuum of a common epileptic condition (Provini et al., 1999). Subsequently, many studies showed that sleep‐related seizures with hyperkinetic automatisms could have also an extra‐frontal origin (Nobili et al., 2004; Proserpio et al., 2011; Ryvlin et al., 2006). During a consensus conference in Bologna, the term NFLE was replaced by SHE to reflect the evidence that seizures are associated with sleep rather than time of day, have characteristic hypermotor features and are not always of frontal lobe origin (Tinuper et al., 2016). It was also stated that although aetiology remains unknown in the majority of patients, it may include structural anomalies such as focal cortical dysplasia and genetic mutations such as CHRNA4, the first recognized epilepsy gene, identified in a large kindred of autosomal dominant SHE (Scheffer et al., 1995; Steinlein et al., 1995). Other genes have since then been recognized including KCNT1 and DEPDC5 (Heron et al., 2012; Picard et al., 2014).

Reviewing the anatomo‐electroclinical data of patients with SHE also clarified seizure pattern subtypes arising from the frontal lobe, and showed that the most highly integrated ictal behaviours tend to emerge from the anterior prefrontal regions, while more elementary motor signs are associated with posterior regions of the frontal lobe (Bonini et al., 2014; Gibbs, Proserpio, et al., 2019). Although sometimes impressive in nature, these seizure manifestations have a cortical correlate that include complex frontal networks as well as subcortical circuitry (Pelliccia et al., 2022; Rheims et al., 2008; Tassinari et al., 2005; Zalta et al., 2020). Distinguishing a frontal from an extra‐frontal onset can be challenging especially in patients with normal brain magnetic resonance imaging. However, certain clues can be useful, including non‐motor seizure semiology (auras), seizure duration and latency between the first detectable movement, usually an awakening, and the onset of hypermotor manifestations (Gibbs et al., 2018; Gibbs et al., 2019). Diagnosis of SHE is based primarily on clinical history and seizure description consisting of obvious and disruptive hypermotor events. Three diagnostic categories are available: (a) witnessed SHE (possible), based on the description of clinical features; (b) video‐documented (clinical) SHE, based on the evaluation of a video‐recorded hypermotor episode; and (c) video‐EEG‐documented (confirmed) SHE, requiring the video‐polygraphic recording of stereotyped events and ictal or interictal epileptiform abnormalities. Because the presence of clear‐cut ictal or interictal epileptiform abnormalities is only observed in a minority of patients, the absence of EEG correlated does not exclude the diagnosis of SHE (Tinuper et al., 2016).

Differential diagnosis is broad, and includes disorders of arousals (DOA), sleep‐related movement disorders, and REM‐sleep behaviour disorder. Differentiation with DOA is often the most challenging due to clinical similarities between both entities. DOA are parasomnic events, characterized by complex, seemingly purposeful behaviours occurring during an incomplete awakening from NREM sleep (American Academy of Sleep Medicine, 2014). DOA often begin in childhood, are of variable frequency and duration and, most importantly, are not stereotypic in nature, as one event will likely be different from another (Castelnovo, Lopez, Proserpio, Nobili, & Dauvilliers, 2018). Careful history taking represents the first step for differentiating SHE and DOA, and this can be sufficient in typical cases. Different questionnaires based on clinical features have been developed as further support tool, though with variable accuracy values (Bisulli et al., 2012; Derry et al., 2006; Loddo et al., 2021; Manni, Terzaghi, & Repetto, 2008). Video‐PSG represents the “gold‐standard” test for diagnosing complex sleep‐related events, but the widespread availability of home‐recording devices also provides a useful and affordable diagnostic instrument, especially if multiple DOA events are captured (Montini, Loddo, Baldelli, Cilea, & Provini, 2021; Nobili, 2009). However, in accordance with the current diagnostic criteria for SHE, if the recorded episodes are minor motor events or PA, the clinical diagnosis may be unreliable (Tinuper et al., 2016). Indeed, recent findings demonstrate that DOAs are characterized not only by major events but also by events of lesser intensity, such as brief arousals called “simple arousal movements” (SAMs; Loddo et al., 2017). These can be difficult to distinguish from minor motor events or PA in patients with SHE. Here, video‐PSG is most helpful as the occurrence of at least one minor event during N3 is highly suggestive for DOA, whereas a major motor event outside N3 is significantly indicative for SHE (Proserpio et al., 2019). Moreover, analysing specific semiological features captured on video‐PSG such as duration, sleep stage at onset, limb involvement, movement progression and behaviours can be useful to differentiate seizure fragment in SHE from SAMs in DOA (Loddo et al., 2020). However, other studies suggest the existence of a possible continuum between the two conditions, mitigating the sharp dichotomy DOA versus SHE (Halász, Kelemen, & Szűcs, 2012; Mutti et al., 2020).

## 8 EPILEPSY AND SLEEP DISORDERS

With a prevalence of epilepsy of 0.7% and the estimate that sleep disorders occur in every third person in his or her lifetime, it is not strange that both conditions may overlap. Sleep disorders often disappear or are successfully treated, but still a major overlap remains. In a large multicentre and long‐term study in Italy, one or more co‐morbidities were found in 26.4% of 1006 PWE. From the 408 reported co‐morbidities, 42.2% appeared to be associated by chance. Unfortunately sleep disorders are not taken as a specific disease, but many of the disorders studied are often accompanied by sleep disorders, for example depressions and other psychiatric diseases (Giussani et al., 2021). In another large study based on questionnaires, sleep disorders were mentioned in adult PWE twice as often when compared with controls (de Weerd et al., 2004). This ratio was far higher in children treated in a tertiary epilepsy centre: major complaints were reported by the parents 12 times more commonly than for healthy children in the same age range (Gutter, Brouwer, & de Weerd, 2013). In both studies, the quality of life (QoL) was lowest in the PWE combined with a sleep disorder. In unselected adults with PWE, 10% had coexisting obstructive sleep apnea (OSA); in a cohort of children and adult patients with drug‐resistant epilepsies, OSA percentages were 20 and 30, respectively (Manni & Terzaghi, 2010). In general, sleep disorders seem to be prevalent in drug‐resistant PWE (Bergmann et al., 2020).

The frequency of primary RLS/PLMS (restless leg syndrome/periodic leg movements) was higher in 98 patients with temporal epilepsy when compared with healthy controls (Geyer, Geyer, Fetterman, & Carney, 2017). In a review of 31 studies (Macêdo, Oliveira, Foldvary‐Schaefer, & Gomes, 2017), the prevalence of insomnia was 28%–51% in PWE when a cut‐off of the Insomnia Severity Index > 15 was used, and 36%–74% when insomnia was diagnosed according the DSM‐IV or the International Classification of Sleep Disorders second edition (American Academy of Sleep Medicine, 2005).

It is clear that epilepsy and sleep have a bi‐directional relationship. The phenomena of epilepsy, ictal and interictal, during the night and antiseizure medication (ASM) have an influence on sleep. Vice versa, sleep itself, but in particular sleep deprivation and sleep disorders, may worsen the severity of epilepsy. As such, these interactions may induce a vicious circle (Eriksson, 2011; Quigg et al., 2021) and have an even more negative influence on the QoL when compared with PWE without a sleep disorder (de Weerd et al., 2004; Gutter et al., 2013). It is often difficult to delineate which factor is most important, the epilepsy or the sleep disorder, for example if a PWE complains of insomnia in addition to seizures. The chosen ASM (e.g. lamotrigine), may induce insomnia, but daytime as well nocturnal seizures combined with frequent interictal EEG abnormalities may also affect sleep (as outlined above). Further, if the PWE is sleepy during the day is this due to seizures during the night, co‐morbid RLS/PLMS/OSA, or is it a side‐effect of the administered ASM?

Recently, a consensus review on the “Standard procedures for the diagnostic pathway of SRE and co‐morbid sleep disorders” was published under the auspices of the European Academy of Neurology, the European Sleep Research Society, and the European chapter of the International League Against Epilepsy (Nobili et al., 2020, 2021). SRE are classified into three groups: (a) sleep‐associated epilepsies (seizures exclusively or almost exclusively from sleep) are SHE, epilepsy with centro‐temporal spikes and the Panayiotopoulos syndrome; (b) sleep‐accentuated epilepsies (consistent extreme potentiation of epileptiform activity during sleep) are ESES, LKS, West syndrome and Lennox–Gastaut syndrome; (c) awakening epilepsies (seizures typically occurring in the period after awakening from sleep) are juvenile myoclonic epilepsy and epilepsy with generalized tonic–clonic seizures alone. A description and value of the recommended aspects of the diagnostic pathway in patients with suspected SRE are given. They are grouped under: clinical history, questionnaires and diaries, tools for capturing the events at home: home video and tools for objective evaluation in the laboratory (e.g. video‐EEG/PSG, actigraphy). Part two describes the recommendations for the diagnosis of SRE together with co‐morbidity with sleep disorders. The diagnostic steps are similar to those in part 1, and include guidelines for management and therapy. The main rule for the diagnostic, management and therapeutic aspects of SRE with co‐morbid sleep disorders is to simultaneously do two complete work‐ups, one for the epilepsy and the other for the co‐morbid sleep disorder (Nobili et al., 2021). For sleep disorders, the ICSD‐3 and recent literature provide description and necessary diagnostic pathways. Further management and decisions about the treatment of epilepsy and co‐morbid sleep disorders are similar to a situation when the disorders are not related to each other (Bruni et al., 2018; Geyer et al., 2017; Nobili et al., 2021; Pornsriniyom et al., 2014; Unterberger et al., 2015; Vignatelli et al., 2006), but the literature on the combination of these diseases is limited. Although not discussed in the standard for sudden unexpected death in epilepsy, knowledge of its prevalence and how to inform the patient is important for all PWE and their doctors, and particularly for patients with nocturnal seizures (Lamberts, Thijs, Laffan, Langan, & Sander, 2012).

## 9 CONCLUSIONS

The impressive contribution of the “European school” in the field of sleep and epilepsy stems from a cultural background that tries to uncover all the information and secrets hidden in scalp‐ and intracerebral‐EEG and polygraphic recordings. To determine if a patient is or is not a carrier of an epileptic syndrome, hours can be spent analysing the traces in search of a paroxysmal anomaly or an alteration of the signal. Starting from these premises of detail and dynamism, the new frontiers of research will continue to explore the bi‐directional interaction between the arousal mechanisms and epileptic susceptibility, but also the impact of epilepsy on the processes of circuit plasticity and memory consolidation that occur during or that are modulated by sleep. Overlap and differentiation between NREM parasomnias, SHE and other sleep pathologies also deserves to be revisited from different perspectives, and perhaps the time is ripe to also include SRE in the list of sleep disorders. Finally, due attention should be devoted to the biological autonomic consequences of epilepsy‐related sleep alterations, and to the acute and long‐term action of ASM on sleep structure.

## AUTHOR CONTRIBUTIONS

Conceptualization: L.N., L.P.; B.F and L.P.D. wrote the first draft of the chapters “The Effects of Sleep on Epilepsy” and the “Effects of Epilepsy on Sleep”; P.H. and I.L. wrote the first draft of the chapters “Epilepsy, Sleep, Brain Plasticity and Epileptogenesis” and “IEDs During NREM Sleep: Impact on Cognition”; S.E. wrote the first draft of the chapter “Circadian Rhythm and Epilepsy”; F.P., P.P., S.G. wrote the first draft of the chapter “From Nocturnal Frontal Lobe Epilepsy to Sleep Hypermotor Epilepsy”; R.M. and A.W. wrote the first draft of the chapter “Epilepsy and Sleep Disorders”; L.N. and L.P. wrote the Introduction, the Conclusions and reviewed all the chapters; all the authors have read and approved the final version of the manuscript.

## Data Availability

Data sharing not applicable ‐ no new data generated
